# Inhibiting Acetylcholinesterase to Activate Pleiotropic Prodrugs with Therapeutic Interest in Alzheimer’s Disease

**DOI:** 10.3390/molecules24152786

**Published:** 2019-07-31

**Authors:** François-Xavier Toublet, Cédric Lecoutey, Julien Lalut, Bérénice Hatat, Audrey Davis, Marc Since, Sophie Corvaisier, Thomas Freret, Jana Sopkova de Oliveira Santos, Sylvie Claeysen, Michel Boulouard, Patrick Dallemagne, Christophe Rochais

**Affiliations:** 1 Normandie Univ, UNICAEN, CERMN, 14000 Caen, France; 2 Univ. Montpellier, CNRS, INSERM, IGF, 34090 Montpellier, France; 3 Normandie Univ, UNICAEN, INSERM, Comete, GIP CYCERON, 14000 Caen, France

**Keywords:** Alzheimer’s disease, prodrug, acetylcholinesterase, 5-HT_4_ receptors, MTDL

## Abstract

Alzheimer’s disease (AD) is a multifactorial neurodegenerative disease which is still poorly understood. The drugs currently used against AD, mainly acetylcholinesterase inhibitors (AChEI), are considered clinically insufficient and are responsible for deleterious side effects. AChE is, however, currently receiving renewed interest through the discovery of a chaperone role played in the pathogenesis of AD. But AChE could also serve as an activating protein for pleiotropic prodrugs. Indeed, inhibiting central AChE with brain-penetrating designed carbamates which are able to covalently bind to the enzyme and to concomitantly liberate active metabolites in the brain could constitute a clinically more efficient approach which, additionally, is less likely to cause peripheral side effects. We aim in this article to pave the road of this new avenue with an in vitro and in vivo study of pleiotropic prodrugs targeting both the 5-HT_4_ receptor and AChE, in order to display a neuroprotective activity associated with a sustained restoration of the cholinergic neurotransmission and without the usual peripheral side effects associated with classic AChEI. This plural activity could bring to AD patients effective, relatively safe, symptomatic and disease-modifying therapeutic benefits.

## 1. Introduction

Acetylcholinesterase (AChE) is the main target of the currently marketed drugs against Alzheimer’s Disease (AD). Inhibiting AChE and the physiological degradation of acetylcholine (ACh), these drugs aim at restoring the cholinergic neurotransmission impaired by the neurodegeneration which induces the cognitive troubles associated with AD. These medicines are controversial today, mainly due to their loss of efficacy over time. AChE, however, could benefit from renewed interest as a target for novel anti-AD drugs for two reasons:

The first concerns the discovery of a new chaperone role played by AChE which would be able to interact, thanks to its peripheral anionic site (PAS), with the β-amyloid (Aβ) peptide and to form neurotoxic aggregates with the latter [1]. Inhibiting this interaction, if possible in a concomitant manner with the catalytic inhibition of AChE, would yield a dual therapeutic benefit against AD.

The second reason is inherent to the explanation which can be given for the loss of efficiency of its inhibitors. The latter can be imputed to the evolution of the disease and to the consequent neurodegeneration which leads to a decrease in the production of AChE, whose inhibition becomes less and less useful over time. However, preserving the functional status of the cholinergic neurons could make AChE an efficient target in AD treatment once again. This explains why a lot of clinical trials assessing potential of novel neuroprotective agents are often done in association with donepezil, the most used AChE inhibitors (AChEI), in order to display a synergistic effect [2]. Alternatively, another approach was recently proposed. It consists of the design of Multi-Target Directed Ligands (MTDL) which are able to both inhibit AChE and to express a neuroprotective effect through an interaction with another target involved in the pathogenesis of AD [3]. Our group recently described, within this framework, the in vitro and in vivo properties of donecopride, the first MTDL able to both inhibit AChE and to activate 5-HT_4_ receptors (5-HT_4_R) with potent antiamnesic effects in animal models of AD [4,5].

**Figure 1:**
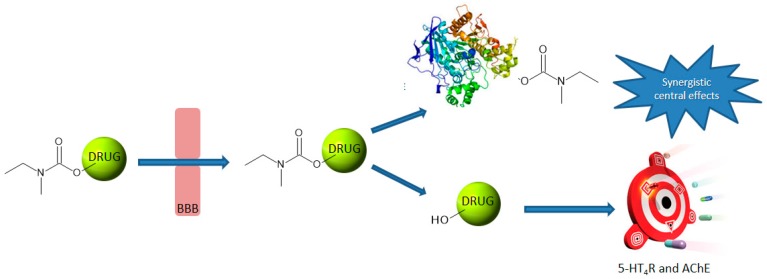
Illustration of the pleiotropic prodrug concept.

These considerations have led to a revival of interest in AChE as an efficient therapeutic target. Furthermore, this enzyme can be used to reduce the peripheral side effects displayed by novel anti-AD drugs. Indeed, the latter can be specifically liberated at the central level through hydrolysis by AChE of brain-penetrating prodrugs. AChE, being inhibited during this hydrolysis, can be considered in this case both as a therapeutic target and as an activating enzyme (Figure 1).

**Figure 2:**
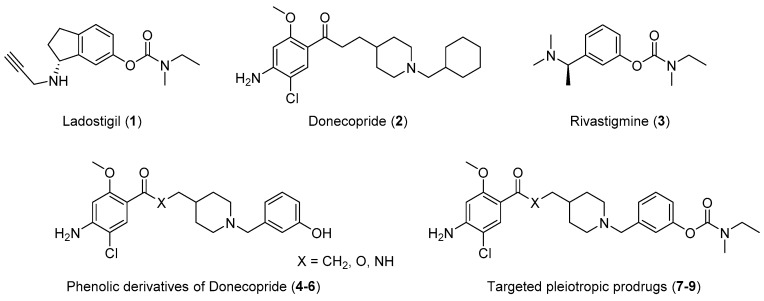
Structure of Ladostigil (**1**), Donecopride (**2**), Rivastigmine (**3**), phenolic derivatives (**4**–**6**) of Donecopride and targeted pleiotropic prodrugs (**7**–**9**).

A unique example of such a pleiotropic anti-AD prodrug is currently reported in literature. Ladostigil (**1**) is a carbamate compound which is able to covalently bind to AChE and to liberate a hydroxy derivative of rasagiline, displaying Monoamine Oxidase (MAO)-B inhibitory activity (Figure 2) [6,7,8,9].

Concerning the present work, we wanted to develop an innovative approach through the design of novel pleiotropic prodrugs which were likely to covalently inhibit AChE and then to liberate active metabolites related to donecopride (**2**) while keeping its potent 5-HT_4_R agonist and Dual Binding Site (DBS) AChE inhibitory activities. These compounds will be designed based on the model of rivastigmine (**3**) with which they are structurally related. Rivastigmine is a carbamate compound which covalently binds and temporarily inhibits AChE. Rivastigmine, which is the only marketed pseudo-irreversible AChEI, consequently restores cholinergic neurotransmission, then alleviating the cognitive symptoms in AD patients. However, the phenolic derivative, released after the carbamoylation of AChE by rivastigmine, is devoid of any therapeutic activity.

The novel phenolic derivatives (**4**–**6**) of donecopride which we propose herein were synthesized and evaluated as potential 5-HT_4_R ligands and DBS AChEI. They were carbamoylated into carbamates (**7**–**9**). The capacity of the latter to inhibit AChE in a covalent manner was investigated. In order to avoid possible peripheral side effects, their lack of other activities was verified. The ability of a selected carbamate to liberate the corresponding phenolic derivative upon AChE-dependent hydrolysis has been studied, as well as its capacity to easily cross the blood brain barrier (BBB). Finally, in vivo tests have been performed in mice in order to potentially highlight, for this pleiotropic prodrug, a procognitive effect.

## 2. Results

### 2.1. Chemistry

**Scheme 1:**
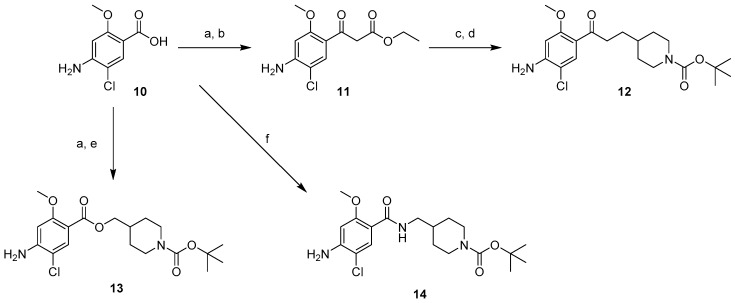
Synthetic pathways for access to compounds **11**–**14**. Reagents and conditions: (**a**) CDI, THF, 8 h, rt; (**b**) KO_2_CCH_2_CO_2_Et, MgCl_2_, THF, 40 °C, 2 h, 42%; (**c**) *N*-Boc 4-iodomethylpiperidine, K_2_CO_3_, DMF, rt, 1 night; (**d**) KPH, EtOH/H_2_O, reflux, 3 h, 56%; (**e**) *N*-Boc 4-hydroxymethylpiperidine, NaH, THF, rt, 3 h, 10%; (**f**) HOBT, EDC.HCl, *N*-Boc 4-aminomethylpiperidine, DMF, rt, 1 night, 15%.

The targeted phenolic derivatives of donecopride (**4**–**6**) and their carbamates (**7**–**9**) were obtained starting from 4-amino-5-chloro-2-methoxybenzoic acid (**10**) in three series: ketone, ester and amide. In the ketone series, the β-ketoester (**11**) was synthesized through the carbonyldiimidazole (CDI) activation of the carboxylic acid group of **10** followed by treatment with the potassium salt of ethylmalonate (Scheme 1). The methylpiperidine moiety was then installed through nucleophilic substitution using *N*-Boc 4-(iodomethyl)piperidine carboxylate at room temperature to avoid the risk of a double substitution, immediately followed by a saponification−decarboxylation sequence with hydroalcoholic potassium hydroxide, which gave **12** [5]. In the ester series, the analog compound **13** was obtained by esterification of **10**, with *N*-Boc 4-hydroxymethylpiperidine, while in the amide series, the analog compound **14** was obtained through a peptidic coupling involving **10** and *N*-Boc 4-aminomethylpiperidine.

**Scheme 2:**
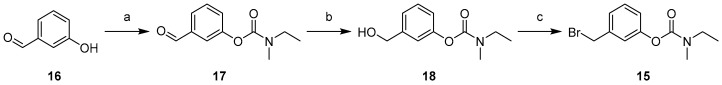
Synthetic pathways for access to compounds **17**–**19**. Reagents and conditions: (**a**) Et(Me)NCOCl, K_2_CO_3_, DMF, 70 °C, 2 h, 87%; (**b**) NaBH_4_, MeOH, 0 °C, 30 min, 84%; (**c**) PBr_3_, Et_2_O, 0 °C, 30 min, 44%.

In parallel, the bromobenzyl carbamate (**15**) was synthesized through the following sequence (Scheme 2). 3-Hydroxybenzaldehyde (**16**) was carbamoylated using ethylmethylcarbamic chloride to yield the carbamate **17** [10]. The aldehyde group of the latter was reduced by NaBH_4_, and the alcohol derivative **18** was brominated by PBr_3_ to give **15**.

**Scheme 3:**
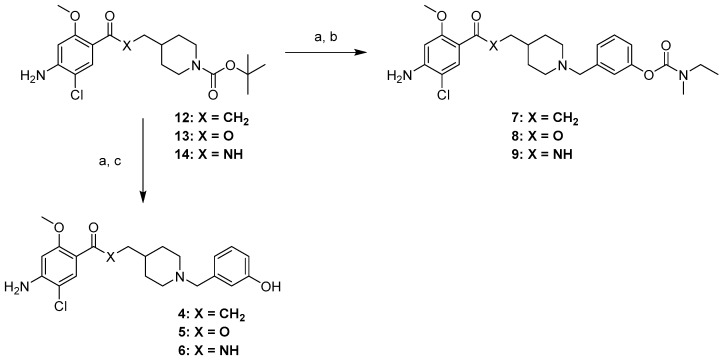
Synthesis pathways for access to compounds **4**–**6** and **7**–**9**. Reagents and conditions: (**a**) TFA, DCM, rt, 1 h; (**b**) **15**, K_2_CO_3_, DMF, 110 °C, 1 h, 49–69%; (**c**) 3-hydroxybenzyl iodide, K_2_CO_3_, DMF, 110 °C, 2 h 30 min, 51–67%.

The targeted compounds were finally obtained through the TFA-*N*-deprotection of **12**–**14**, immediately followed by another nucleophilic substitution with 3-iodomethylphenol [11] to yield phenols **4**–**6** or with compound **15** to form carbamates **7**–**9** (Scheme 3).

**Scheme 4:**
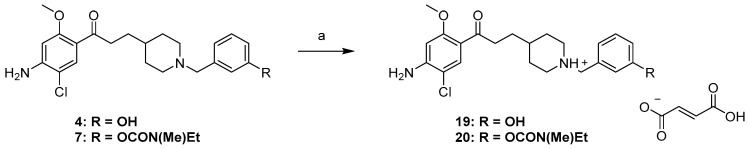
Synthesis pathways for access to **19**, **20**. Conditions and reagents: (**a**) fumaric acid, iPrOH, reflux, 1 h 15 min, 66–68%.

Having been selected for in vivo studies, the fumaric acid salts of phenol **4** and its carbamate **7**, (**19**, **20**) were synthesized using fumaric acid in iPrOH (Scheme 4).

### 2.2. In Silico Results

**Figure 3:**
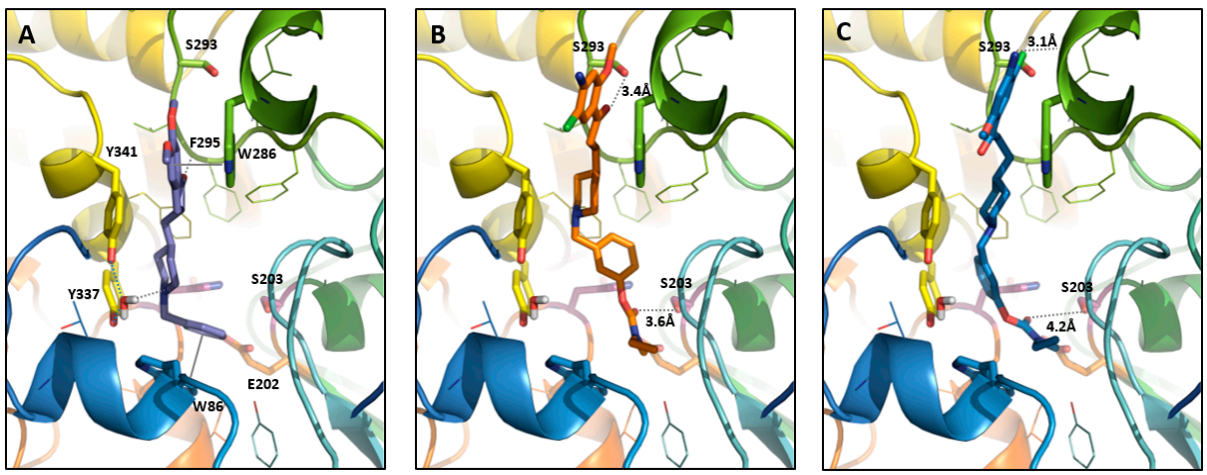
Donepezil position from the X-ray structure (**A**) compared to the compound **7** positioned in (*h*)AChE binding sites using the docking studies, cluster 1 (**B**) and cluster 2 (**C**). The compound and the selected side chains of the binding site residues are in stick and the protein in ribbon representation. This figure was made with PyMOL (DeLano Scientific, 2002, San Carlo, USA).

During in silico studies, we evaluated whether carbamate derivative **7** could access the (*h*)AChE catalytic triad, specially the Ser203 residue playing a crucial role during carbamoylation. According to the proposed mechanism of rivastagmine carbamoylation [12], the carbamate-to-serine grouping approach starts via a hydrogen bond between the oxygen atom of the carbamate carbonyl group and the hydroxy group of Ser203. Therefore, during the docking study of compound **7** into the (*h*)AChE active site (PDB code: 4EY7 [13]), performed using the Gold software (v5.7.2, Cambridge Crystallographic Data Center, CCDC), a hydrogen bond constraint between carbamate carbonyl and hydroxy group of Ser203 was applied. In parallel, the Ser203 side chain was kept flexible during the docking. The generated poses of compound **7** with scores between 85.44 and 100.64 could be divided in two major clusters which differ principally in the position of the methoxy phenyl moiety and phenyl one. In cluster 1 (see Figure 3B), the carbonyl group established a hydrogen bond with Ser293, and in cluster 2, a π-stacking between compound **7** phenol ring and the Tyr 337 one was observed, as well as a possible interaction through a hydrogen bond between the NH_2_ group of compound 7 and C=O of the Trp286 backbone (see Figure 3C).

Compound **7** docks in the AChE active site with the carbamate group in proximity of Ser203, as imposed by the constraint, but it is positioned much higher in the AChE binding site in two clusters compared to the donepezil and/or the donecopride (Figure 3) [5,6,7,8,9,10,11,12,13]. Consequently, according to our molecular modeling results, compound **7** loses the characteristic interactions of the donecopride ligands family, i.e.,: i) interaction between NH^+^ of piperidine ring through the water molecule lying between the hydroxy groups of two tyrosines (Tyr341 and Tyr337); ii) interaction via C=O group with the backbone NH of Phe295; and iii) the methoxyphenyl moiety is placed a little high but stacking with Trp286 remains possible. On the other hand, the C=O group of compound **7** establishes a new interaction through a hydrogen bond with the hydroxy group of Ser293 in cluster 1. So, the ‘donecopride’ part attached to carbamate of compound **7** brings few interactions in addition to carbamate group ones, and the interaction with the AChE binding site is realized mainly thanks to the carbamate group.

### 2.3. In Vitro Results

#### 2.3.1. AChE Inhibition and 5-HT_4_R Binding

All the synthesized phenolic derivatives and their carbamates were evaluated as potential inhibitors of human AChE, using the Ellman assay [14], as well as potential ligands for human 5-HT_4_R using a radioligand displacement assay [15]. In these tests, Donepezil and Rivastigmine (**3**) were used as reference AChEI, and RS67333 as a reference 5-HT_4_R agonist [5]. The results are depicted in Table 1.

All the carbamates appeared able to inhibit AChE with IC_50_ values in the same range as rivastigmine. The keto derivative **7** exhibited the best activity with an IC_50_ value of 4.15 µM which was recovered with its fumaric salt, **20**. The phenolic derivatives in the ester (**5**) and amide (**6**) series appeared to be devoid of any AChE inhibitory activities, unlike their analog **4** in keto series displaying a noticeable AChEI activity which turned to be even better for its fumaric salt **19** (respectively, IC_50_ value of 148 and 72 nM).

Concerning 5-HT_4_R affinity, carbamates **7**–**9** and **20** appeared to be devoid of such activity, while their phenolic analogs **4**–**6** and **19** appeared to be potent ligands with K*i* values in the same range as RS67333 (5 nM), or even decreased for the ester derivative **5** (0.6 nM).

#### 2.3.2. Pharmacological Profile Results

**Figure 4:**
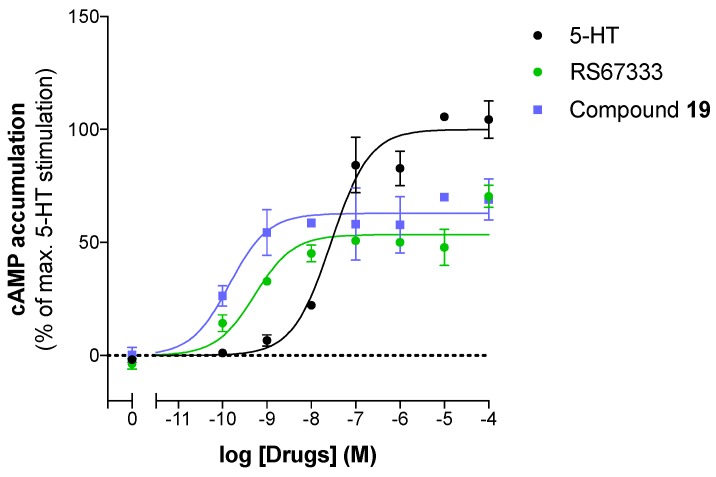
Pharmacological profile of RS67333 and compound **19**. Representative experiment illustrating agonist activities towards (*h*)5-HT_4(a)_ receptors.

The pharmacological profile of the selected phenolic derivative **19** was first established towards (*h*)5-HT_4_R. It acts as a partial agonist in a similar manner as RS67333 (Figure 4 and Table 2).

**Figure 5:**
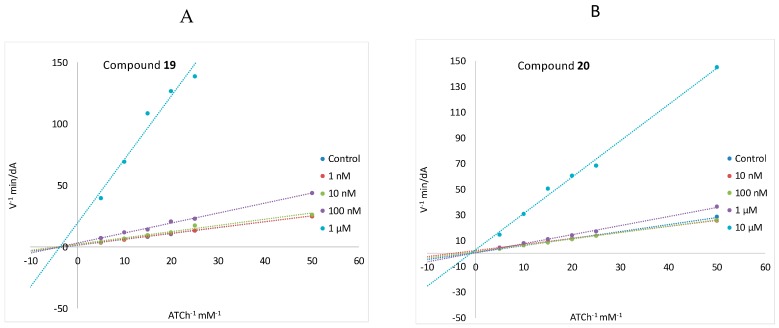
Lineweaver-Burk plots of inhibition kinetics show that **19** acts as a non-competitive AChE inhibitor (**A**) and **20** as a mixed-type AChE inhibitor (**B**).

On the other hand, the mechanism of AChE inhibition for compounds **19** and **20** was evaluated by means of a kinetic study, the results of which are reported in typical Lineweaver-Burk plots. Phenol **19** acts as a non-competitive inhibitor, accounting for a possible interaction of **19** with the PAS of the enzyme (Figure 5A). At the same time, the carbamate **20** showed a mixed-type inhibition, illustrated by a typical Lineweaver-Burk plot similar to those obtained with the covalent AChEI, rivastigmine (Figure 5B) [18].

#### 2.3.3. Brain Penetration

The ability of carbamate **20** to cross the BBB was assessed using a parallel artificial membrane permeability assay experiment. The compound was classified among the compounds having good brain penetration with logPe = −4.39 (Pe represents the PAMPA effective permeability coefficient). The test was performed using a good (corticosterone) and weak brain-penetrating reference (theophylline), respectively (Table 3).

#### 2.3.4. AChE-Dependent Decarbamoylation

The in vitro decarbamoylation of **20** in the presence of AChE was established according to a new analytical method developed by Alix et al. [19]. A large excess of *Electrophorus electricus* (*eel*)AChE was added to a solution of **20** in a phosphate-buffered saline (PBS) buffer (pH 7.4) at 25 °C. After 24 h of incubation, the solution was extracted by ethyl acetate, evaporated and solubilized in acetonitrile, and then analyzed by UPLC and LCMS. Owing to its high homology with the active-site sequence of the human AChE and its commercial availability, (*ee*)AChE is commonly used for in vitro assays and was chosen for this study.

**Figure 6:**
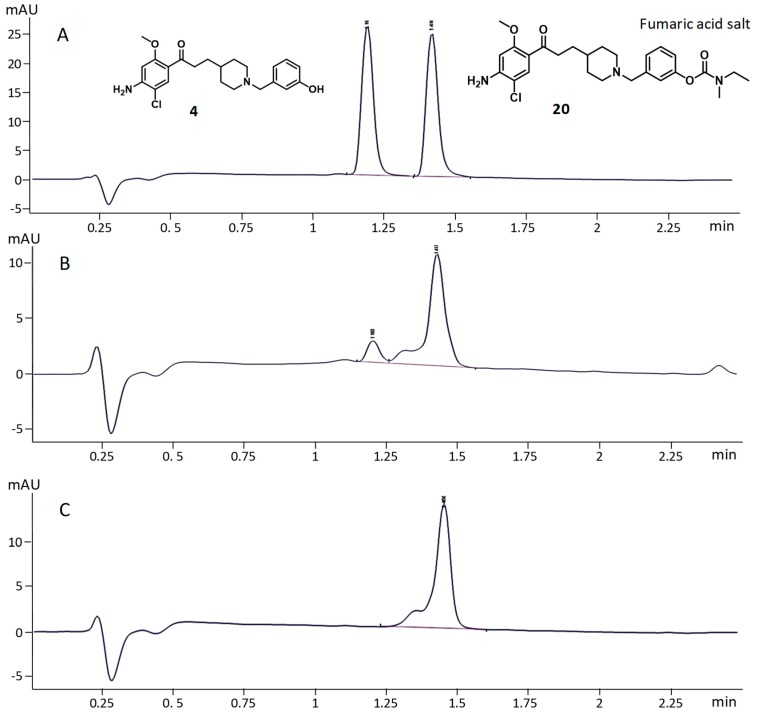
Decarbamoylation of **20** by (*ee*)AChE; (**A**): **20** (5 µM) and **4** (5 µM) without (*ee*)AChE; (**B**): **20** (5 µM) in the presence of 250 U/mL (*ee*)AChE (**C**): **20** (5 µM) without (*ee*)AChE (negative control).

According to ULPC analysis and on the basis of the retention times of **4** and **20** without (*ee*)AChE (Figure 6A), the first result showed a decarbamoylation of **20** and release of **4** (Figure 6B). The control experiment in a PBS buffer without (*ee*)AChE, under the same reaction conditions, was also performed to ensure that in the absence of (*ee*)AChE, **20** was not chemically converted into **4** (Figure 6C). Rivastigmine was used as a positive control. These results were confirmed by mass analysis.

### 2.4. In Vivo Results

Some in vivo investigations have been performed on the selected carbamate **20** as a potential pleiotropic prodrug.

#### 2.4.1. Pharmacological Screening

None of the dose tested (1, 10 and 100 mg/kg) for compound **20** showed serious side effects (Table 4). Indeed, even though only for the highest tested dose, tremors and following hypoactivity phase were noticed, all symptoms were absent after 24 h, suggesting a LD_50_ that is significantly higher than 100 mg/kg.

#### 2.4.2. Spontaneous Locomotor Activity

**Figure 7:**
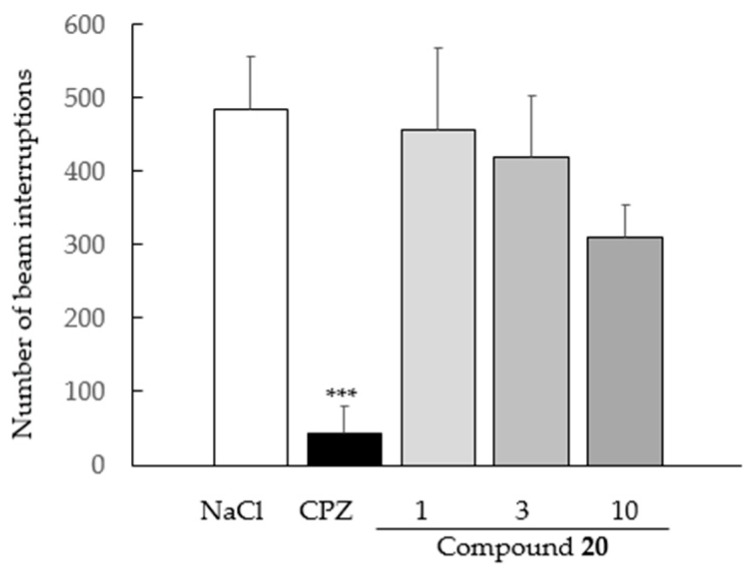
Effect of compound **20** on spontaneous locomotor activity. Data are expressed as the mean ± standard error of mean (SEM, *n* = 8 per group). Drugs were administered intraperitoneally (IP) 30 min before the behavioral test. Compound **20**: 1–3–10 mg/kg; CPZ: chlorpromazine 3 mg/kg (*** *p* < 0.05 versus NaCl, SNK test).

Except for chlorpromazine-treated animals (used as reference drug, *p* < 0.0001), none of the tested doses of compound **20** (1, 3 and 10 mg/kg) statically modified spontaneous locomotor activity in comparison to NaCl-treated control animals (Figure 7). ANOVA revealed a group effect (F_(4,37)_ = 6.495, *p* = 0.0005).

#### 2.4.3. Spontaneous Alternation Deficit

**Figure 8:**
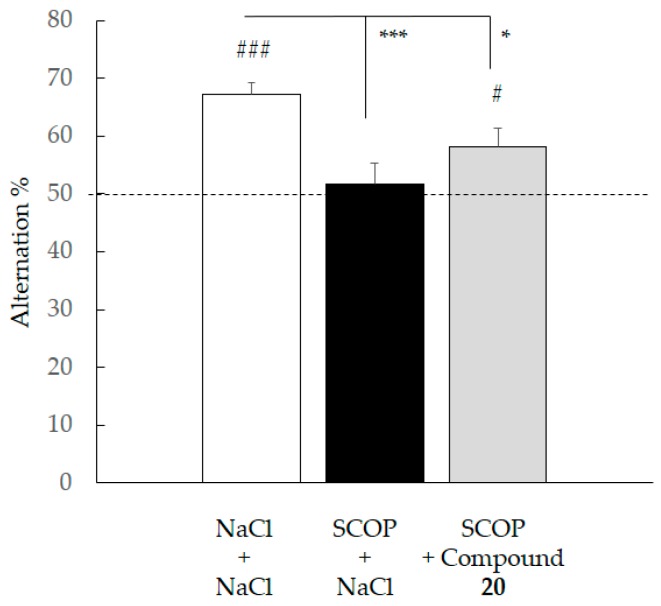
Effect of compound **20** on scopolamine impairment during the spontaneous alternation test. Data are expressed as the mean ± standard deviation (SEM, *n* = 8–10 per group) NaCl and compound 20 (1 mg/kg) were administered IP 30 min before the test, scopolamine (SCOP, 0.5 mg/kg)) was administered SC 20 min before the test. (# *p* < 0.05, ### *p* < 0.001 versus 50%; univariate *t*-test). (* *p* < 0.05, *** *p* < 0.001 versus other groups, SNK test).

**Figure 9:**
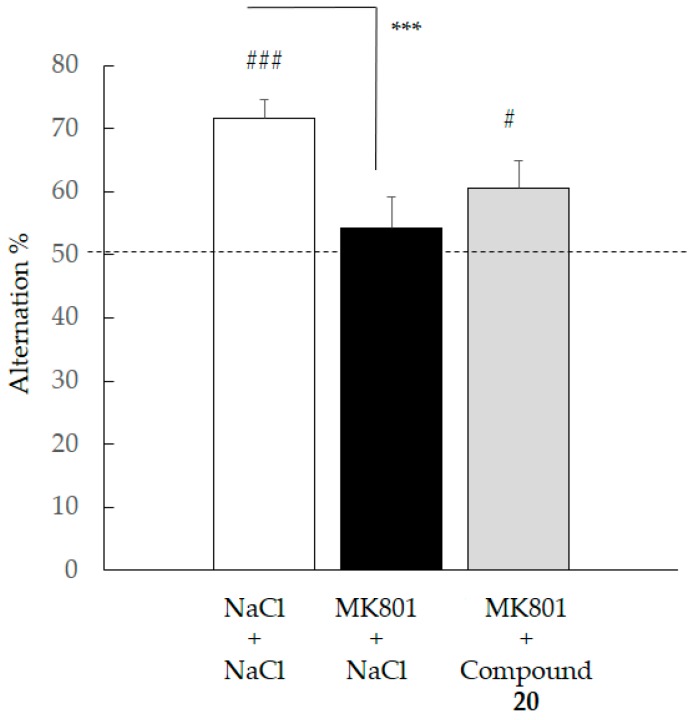
Effect of compound **20** on MK 801 induced impairment during the spontaneous alternation test. Data are expressed as the mean ± standard deviation (SEM, *n* = 8–10 per group). NaCl and compound **20** (1 mg/kg) were administered IP 30 min before the test, MK801 (0.1 mg/kg) was administered SC 20 min before the test. (# *p* < 0.05, ### *p* < 0.001 versus 50%; univariate *t*-test). (*** *p* < 0.05 versus MK801, SNK test).

We then decided to evaluate the in vivo efficacy of compound **20** in cognition models. Whatever the pharmacological agent used to induce a cognitive deficit (scopolamine – SCOP or dizocilpine, MK801), ANOVA of percentage of spontaneous alternation revealed a group effect (respectively, F(2,23) = 7.281; *p* = 0.0036 and F(2,25) = 4.778; *p* = 0.0175) (Figure 8 and Figure 9). In both cases, the control group displayed an alternation percentage significantly higher than animals receiving either SCOP or MK801 plus NaCl, demonstrating a pharmacologically-induced cognitive deficit (SNK, *p* < 0.001). Further, while the control group showed higher spontaneous alternation than animals receiving SCOP + compound **20** (SNK, *p* < 0.05), no statistical difference was observed compared to animals receiving MK801 + compound 20 (SNK, *p* = 0.0621). Additionally, a univariate *t*-test revealed that in the two conditions (SCOP and MK801), only the control group and animals having received compound **20** displayed an alternation percentage which was significantly different from the value of the chance level (i.e., 50%). Hence, those results point to the ability of compound **20** to offset—at least partially—working memory deficits, whether they result from cholinergic or glutamatergic neurotransmission alteration. Interestingly, these anti-amnestic effects are effective at a relatively low dose with respect to the estimated LD_50_.

## 3. Discussion

An in vitro evaluation of the three phenolic derivatives of donecopride **4**–**6** led us to select **4** in the keto series on the basis of its AChE inhibitory activity (IC_50_ = 149 nM); such activity appeared to be absent in **5** and **6**. The 5-HT_4_R affinity of **4** (Ki = 5.1 nM) appeared to be slightly less potent than its ester analogue (K*i* = 0.6 nM), but was preferred due to its dual activity. The fumaric acid salt of **4**, **19**, which maintains the dual activity, further expresses a partial 5-HT_4_R agonist pharmacological profile and a non-competitive-type AChE inhibitory profile. Consequently, it would act as a DBS AChEI. The carbamate **7**, as well as its fumaric acid salt **20**, derived from **4**, are devoid of any activity towards 5-HT_4_R in a similar manner as **8** and **9**. This lack of activity was sought in order to reduce the occurrence of possible peripheral side effects. Compounds **7** and **20** further inhibited AChE with a similar intensity as rivastigmine (IC_50_ = 4.1 μM). The kinetic study of **20**, furthermore, established its covalent binding to the enzyme, and an analytic study demonstrated its concomitant AChE-dependent hydrolysis and the release of the corresponding phenolic derivative **4**. Finally, the capacity of **19** to easily cross the BBB was attested using the PAMPA model.

On the basis of these predictive pharmacodynamic and pharmacokinetic profiles, **20** was selected for two in vivo studies aiming at demonstrating its potential antiamnesic effect in two different models of spontaneous alternation deficit in mice using either scopolamine or MK801 to induce cognitive impairments.

These results support our expected mechanism of action and afford preliminary proof of concept of an in vivo release of a phenolic derivative (from carbamate precursor). Hence, having irreversibly blocked acetylcholinesterase during its release from carbamate, the phenolic derivative would then be capable of modulating our serotoninergic receptors of interest (5-HT_4_ receptors). Further studies will be needed to validate this, but some data from the literature reinforce this hypothesis. In particular, some studies in rodents have shown the ability of rivastigmine (which has a pharmacodynamic profile close to that of our compound **20**) to counterbalance scopolamine-induced memory performance deficit in various tests [20,21]. Interestingly, this same rivastigmine, used under similar conditions, proved to be ineffective in improving memory deficits induced by MK 801 [22]. Thus, the superiority in the MK801 model of our compound relative to rivastigmine may be related to the release of the phenolic derivative having affinity and significant activity towards 5-HT_4_ receptors.

## 4. Materials and Methods

### 4.1. Chemistry

#### 4.1.1. General Methods

All chemical reagents and solvents were purchased from commercial sources and used without further purification. Melting points were determined on a STUART SMP50 melting point apparatus. ^1^H, ^13^C and ^19^F NMR spectra were recorded on a BRUKER AVANCE III 400MHz with chemical shifts expressed in parts per million (in chloroform-*d*, methanol-*d*_4_ or DMSO-*d*_6_) downfield from TMS as an internal standard and coupling in Hertz. IR spectra were recorded on a Perkin-Elmer BX FT-IR apparatus using KBr pellets. High resolution mass spectra (HRMS) were obtained by electrospray on a BrukermaXis. The purities of all tested compounds were analyzed by LC−MS, with the purity all being higher than 95%. Analyses were performed using a Waters Alliance 2695 as separating module (column XBridge C18 2.5 μM/4.6 × 50 mM) using the following gradients: A (95%)/B (5%) to A (5%)/B (95%) in 4.00 min. This ratio was hold during 1.50 min before returning to initial conditions in 0.50 min. Initial conditions were then maintained for 2.00 min (A = H_2_O, B = CH_3_CN, each containing HCOOH: 0.1%). MS were obtained on a SQ detector by positive ESI.

#### 4.1.2. Synthesis of Compounds (**11**–**18**)

*Ethyl 3-(4-amino-5-chloro-2-methoxyphenyl)-3-oxopropanoate* (**11**) [5]. To a mixture of 4-amino-5-chloro-2-methoxybenzoic acid (**10**) (2.0 g, 9.92 mmol, 1.0 equiv) and carbonyldiimidazole (1.77 g, 10.91 mmol, 1.1 equiv), dry THF (100 mL) (caution: gas evolution) was added. The reaction mixture was stirred at room temperature for 8 h, and then potassium 3-ethoxy-3-oxopropanoate (2.03 g, 11.90 mmol, 1.2 equiv) and MgCl_2_ (1.13 g, 11.90 mmol, 1.2 equiv) were added portion-wise. The reaction mixture was stirred at 40 °C for 2 h. Evaporation of the solvent provided a residue that was diluted in water. The mixture was extracted with DCM. The organic layer was washed with water, aqueous NaHCO_3_ and brine; then, it was dried over MgSO_4_. Evaporation of the solvent provided a crude product that was purified by column on silica, eluting with a gradient of 20–30% EtOAc/cyclohexane to give (1.14 mg, 42% isolated yield) as a white solid **6**. ^1^H NMR (MeOD, 400 MHz, 298K) *δ* 7.91 (s, 1H), 6.23 (s, 1H), 4.59 (s, 2H), 4.17 (q, 2H, *J* = 7.2 Hz), 3.87 (s, 2H), 3.82 (s, 3H), 1.24 (t, 2H, *J* = 7.2 Hz); ^13^C NMR (MeOD, 100 MHz, 298 K) *δ* 189.8, 168.8, 160.0, 148.9, 132.6, 117.4, 111.8, 97.2, 61.0, 55.6, 50.6, 14.3.

*Tert-butyl 4-[3-(4-amino-5-chloro-2-methoxyphenyl)-3-oxopropyl]piperidine-1-carboxylate* (**12**) [4]. To a DMF (30 mL) solution of compound **11** (860 mg, 3.17 mmol, 1.0 equiv) and *N*-Boc 4-iodomethylpiperidine (1.24 g, 3.81 mmol, 1.2 equiv) was added K_2_CO_3_ (877 mg, 6.35 mmol, 2.0 equiv), and the mixture was stirred at room temperature for 1 night. The reaction mixture was diluted with water, and the product was extracted with EtOAc. The combined organic layer was washed with water and brine and then dried over MgSO_4_. Evaporation of the solvent gave a crude mixture (2.04 g, 4.34 mmol), which was directly dissolved in EtOH (87 mL). To the solution, H_2_O (17 mL) and KOH (1.1 g, 19.6 mmol, 4.5 equiv) were added. The reaction mixture was refluxed for 3 h. EtOH was evaporated. The mixture was diluted with H_2_O and extracted with EtOAc. The organic layer was washed with H_2_O and brine. Evaporation of the solvent provided a crude product, which was purified by column on silica, eluting with a gradient of 0–20% of EtOAc/DCM to give the compound **6** as a colorless solid (710 mg, 56% isolated yield). ^1^H NMR (CDCl_3_, 400 MHz, 298 K) *δ* 7.79 (s, 1H), 6.26 (s, 1H), 4.46 (s, 2H), 4.16–3.98 (m, 2H), 3.85 (s, 3H), 2.94–2.87 (m, 2H), 2.73–2.60 (m, 2H), 1.71–1.63 (m, 2H), 1.62–1.56 (m, 2H), 1.45 (s, 9H), 1.40 (m, 1H), 1.16–1.04 (m, 2H); ^13^C NMR (CDCl3, 100 MHz, 298 K) *δ* 198.9, 159.6, 155.0, 147.8, 132.4, 119.0, 111.4, 97.7, 79.3, 55.8, 44.0, 40.8, 35.9, 32.2, 31.3, 28.6 (3C).

*Tert-butyl 4-[(4-amino-5-chloro-2-methoxybenzoyl)oxymethyl]piperidine-1-carboxylate* (**13**). To a suspension of **10** (487 mg, 2.42 mmol) in dry THF (55 mL) was added CDI (431 mg, 2.66 mmol). The mixture was stirred at room temperature for 24 h, and then a solution of *N*-Boc 4-hydroxymethylpiperidine (520 mg, 2.42 mmol) in dry THF (10 mL) and NaH 95% (244 mg, 9.66 mmol) were added. This new mixture was stirred 3 h at room temperature and then concentrated under reduced pressure. The residue was dissolved in AcOEt, washed with water, dried over MgSO_4_ and concentrated in vacuo. The crude product was then purified by chromatography on an alumine gel column (gradient of elution: DCM (100%) to AcOEt (100%)). Compound **7** was obtained as a colorless oil (100 mg, 10% isolated yield). ^1^H NMR (CDCl_3_, 100 MHz, 298 K) *δ* 7.78 (s, 1H), 6.28 (s, 1H), 4.53 (s, 1H), 4.12 (m, 2H), 4.08 (d, 2H, *J* = 6.4 Hz), 3.81 (s, 3H), 2.78–2.64 (m, 2H), 1.89 (m, 1H), 1.79–1.69 (m, 2H), 1.44 (s, 9H), 1.31–1.18 (m, 2H, H); ^13^C NMR (CDCl_3_, 100 MHz, 298 K) *δ* 164.7, 160.4, 154.9, 148.0, 133.3, 110.0, 109.6, 98.3, 79.5, 68.4, 56.2, 43.6 (2C), 35.8, 28.9 (2C), 28.6 (3C); MS (*m*/*z*) [M + H]^+^ 299.53/301.59; HRMS (*m*/*z*) calcd. for C_19_H_27_ClN_2_NaO_5_ [M + H]^+^ 421.1506, found 421.1508; IR (neat, cm^−1^) ν_max_ 3445, 3327, 3213, 2975, 2925, 2852, 1695, 1624, 1602, 1421, 1237, 1173.

*Tert-butyl 4-[[(4-amino-5-chloro-2-methoxybenzoyl)amino]methyl]piperidine-1-carboxylate* (**14**) [5]. To a solution of **10** (1.19 g, 5.92 mmol) in DMF (38 mL) were added Et_3_N (825 μL, 5.92 mmol) and *tert*-butyl 4-(aminomethyl)piperidine-1-carboxylate (1.27 g, 5.92 mmol). The mixture was cooled to −5 °C, and HOBT (798 mg, 5.92 mmol) and EDCI·HCl (1.14 g, 5.92 mmol) were then added. The mixture was then stirred for 1 night. After dilution with water, the solution was extracted 3 times with AcOEt. The organic layers were combined, washed 3 times with NaHCO_3_, and once with brine, dried over MgSO_4_, filtered and concentrated under reduced pressure. The crude residue was purified on silica gel (gradient of elution: DCM to DCM/AcOEt 9/1) to provide 350 mg of desired product **8**, a beige powder (15% isolated yield). ^1^H NMR (CDCl_3_, 100 MHz, 298 K) *δ* 8.10 (s, 1H), 7.75 (t, 1H, *J* = 5.3 Hz), 6.26 (s, 1H), 4.39 (br s, 2H), 4.20–4.03 (m, 2H), 3.90 (s, 3H), 3.32 (br s, 2H), 2.74–2.62 (m, 2H), 1.70–1.61 (m, 3H), 1.44 (s, 9H), 1.25–1.10 (m, 2H); ^13^C NMR (CDCl_3_, 100 MHz, 298 K) *δ* 164.8, 157.5, 154.9, 146.8, 133.2, 112.5, 111.7,97.9, 79.5, 56.3, 45.1, 44.1 (2C), 36.6, 30.0 (2C), 28.6 (3C);

*(3-Formylphenyl) N-ethyl-N-methylcarbamate* (**17**). K_2_CO_3_ (6.79 g, 49.13 mmol, 3.0 equiv) was added to a solution of 3-hydroxybenzaldehyde (**16**) (2.0 g, 16.38 mmol, 1.0 eq) in DMF (70 mL) with ice bath cooling. After 5 min, the ethylmethylcarbamic chloride (2.7 mL, 24.57 mmol, 1.5 equiv) was added to the mixture. The solution was then stirred for 2 h at 70 °C. After concentrating the mixture in vacuo, the crude product was dissolved with EtOAc, then the organic layer was washed with brine, dried over MgSO_4_ and concentrated in vacuo. The crude product was purified by chromatography on silica gel column (cyclohexane/EtOAc, gradient 100:0 to 70:30) and concentrated under reduced pressure to afford the compound **10** as a yellowish oil (2.96 g, 87% isolated yield). ^1^H NMR (DMSO, 400 MHz, 363 K) *δ* 10.02 (s, 1H), 7.76 (td, 1H, *J* = 7.8, 1.2 Hz), 7.65 (ddd, 1H, *J* = 2.4, 1.2, 0.4 Hz), 7.61 (dd, 1H, *J* = 7.8, 0.4 Hz), 7.46 (ddd, 1H, *J* = 7.8, 2.4, 1.2 Hz), 3.41 (q, 2H, *J* = 7.1 Hz), 3.00 (s, 3H), 1.19 (t, 3H, *J* = 7.1 Hz); ^13^C NMR (DMSO, 100 MHz, 363 K) *δ* 191.6, 152.8, 151.6, 137.2, 129.6, 127.2, 125.6, 121.4, 43.2, 33.3, 12.0; MS (*m*/*z*) [M + H]^+^ 208.40; HRMS (*m*/*z*) calcd. for C_11_H_14_NO_3_ [M + H]^+^ 208.0974, found 208.0976; IR (neat, cm^−1^) ν_max_ 2974, 1721, 1234.

*[3-(Hydroxymethyl)phenyl] N-ethyl-N-methylcarbamate* (**18**). To a solution of **17** (1.02 g, 4.94 mmol, 1.0 equiv) in MeOH (50 mL) was reduced with NaBH_4_ (561 mg, 14.82 mmol, 3.0 equiv) at 0 °C for 30 min. The solvent was removed in vacuo and the crude product was dissolved with EtOAc; then, the organic layer was washed with brine, dried over MgSO_4_ and concentrated in vacuo. The compound **11** was obtained as a yellow oil (865 mg, 84% yield). ^1^H NMR (DMSO, 400 MHz, 363 K) *δ* 7.30 (t, 1H, *J* = 7.8 Hz), 7.14 (d, 1H, *J* = 7.8 Hz), 7.07 (s, 1H), 6.9 (ddd, 1H, *J* = 7.8, 1.6, 0.6 Hz), 4.94 (t, 1H, *J* = 5.7 Hz), 4.52 (d, 2H, *J* = 5.7 Hz), 3.39 (q, 2H, *J* = 7.1 Hz), 2.97 (s, 3H), 1.17 (t, 3H, *J* = 7.1 Hz); ^13^C NMR (DMSO, 100 MHz, 363 K) *δ* 153.2, 151.1, 143.7, 128.2, 122.3, 119.2, 119.01, 62.2, 43.0, 33.2, 12.1; MS (*m*/*z*) [M + H]^+^ 210.47; HRMS (*m*/*z*) calcd. for C_11_H_15_NNaO_3_ [M + Na]^+^ 232.0950, found 232.0948; IR (neat, cm^−1^) ν_max_ 3419, 2975, 2937, 2877, 1704, 1436, 1402, 1238, 1170, 1088, 1030, 758, 691.

*[3-(Bromomethyl)phenyl] N-ethyl-N-methylcarbamate* (**15**). To a solution of **18** (1.25 g, 5.97 mmol, 1.0 equiv) in Et_2_O (30 mL) was added phosphorus tribromide (0.57 mL, 5.97 mmol, 1 equiv) at 0 °C. After a stirring of 30 min, the supernatant ether solution was decanted, washed with brine and dried over anhydrous MgSO_4_. The solvent was evaporated under reduced pressure. After purification by chromatography on silica gel column (cyclohexane/EtOAc, gradient 80:20), the compound **12** was obtained as a colorless oil (243 mg, 33% isolated yields). ^1^H NMR (DMSO, 400 MHz, 363 K) *δ* 7.36 (t, 1H, *J* = 7.8 Hz), 7.28 (dd, 1H, *J* = 7.8, 1.3 Hz), 7.21 (t, 1H, *J* = 7.8 Hz), 7.16 (ddd, 1H, *J* = 7.8, 2.3, 1.3 Hz), 4.67 (s, 2H), 3.39 (q, 2H, *J* = 7.1 Hz), 2.98 (s, 3H), 1.17 (t, 3H, *J* = 7.1 Hz); ^13^C NMR (DMSO, 100 MHz, 363 K) *δ* 153.0, 151.1, 138.8, 128.9, 125.2, 121.8, 121.1, 43.1, 33.3, 33.0, 12.1; MS (*m*/*z*) [M + H]^+^ 272.44/274.47; (*m*/*z*) calcd. for C_11_H_15_BrNO_2_ [M + H]^+^ 272.0286, found 272.0287/274.0267; IR (neat, cm^−1^) ν_max_ 3438.5, 2972, 2931, 1719, 1395, 1248, 1161, 691.

#### 4.1.3. General Procedure for the Synthesis of Compounds (**4**–**6**)

Procedure A. To a stirred solution of *tert*-butyl piperidine-1-carboxylate derivative (1.0 equiv.) in DCM (20 mL/mmol) was added TFA (2 mL/mmol). The resulting mixture was stirred at room temperature for 1h. Removal of the solvent under vacuum afforded the crude product, which was directly engaged in the next step. The residue obtained (1.0 equiv) was dissolved in DMF (10 mL/mmol) and 3-(iodomethyl)phenol (1.3 equiv) and K_2_CO_3_ (10.0 equiv) were added. The resulting mixture was stirred at 110 °C for 2 h 30 min, then concentrated in vacuo. Ethyl acetate was added, and the organic layer was washed several times with brine, dried over MgSO_4_ and concentrated in vacuo. The crude product was purified by flash chromatography on silica gel column (DCM/MeOH, gradient 100:0 to 90:10) and concentrated under reduced pressure to afford alkylated compound.

*1-(4-Amino-5-chloro-2-methoxyphenyl)-3-[1-[(3-hydroxyphenyl)methyl]-4-piperidyl]propan-1-one* (**4**). Following procedure A with compound **12** (300 mg, 0.76 mmol). We obtained compound **4**, as a white powder (150 mg, 51% isolated yield). ^1^H NMR (MeOD, 400 MHz, 298 K) *δ* 7.64 (s, 1H), 7.12 (t, 1H, *J* = 8.1 Hz), 6.79–6.75 (m, 1H), 6.76 (s, 1H), 6.69 (m, 1H), 6.44 (s, 1H), 3.85 (s, 3H), 3.42 (s, 2H), 2.93–2.86 (m, 4H), 2.15 (s, 2H), 2.03–1.94 (m, 2H), 1.73–1.67 (m, 2H), 1.58–1.51 (m, 2H), 1.32–1.22 (m, 3H); ^13^C NMR (MeOD, 100 MHz, 298 K) *δ* 201.1, 161.7, 158.5, 151.7, 139.7, 132.9, 130.2, 122.0, 117.9, 117.7, 115.3, 111.6, 98.1, 64.4, 56.1, 54.8 (2C), 41.7, 36.8, 32.8 (2C), 32.7; MS (*m*/*z*) [M + H]^+^ 403.60/405.61; HRMS *m*/*z* calcd. for C_22_H_28_N_2_O_3_Cl [M + H]^+^ 403.1788, found 403.1801; m.p. 162.0 °C; IR (neat, cm^−1^) ν_max_ 3469, 3321, 3204, 2920, 2853, 1624, 1580, 1450, 1216.

*[1-[(3-Hydroxyphenyl)methyl]-4-piperidyl]methyl 4-amino-5-chloro-2-methoxybenzoate* (**5**). Following procedure A with compound **13** (95 mg, 0.25 mmol). We obtained compound **5**, as a brown oil (65 mg, 67% isolated yield). ^1^H NMR (MeOD, 400 MHz, 298 K) *δ* 7.70 (s, 1H), 7.13 (m, 1H), 6.81–6.77 (m, 2H), 6.69 (m, 1H), 6.46 (s, 1H), 4.08 (d, 2H, *J* = 4.0 Hz), 3.08 (s, 3H), 3.47 (s, 3H), 3.00–2.93 (m, 2H), 2.11–2.02 (m, 2H), 1.82–1.72 (m, 3H), 1.50–1.37 (m, 2H); ^13^C NMR (MeOD, 100 MHz, 298 K) *δ* 166.7, 162.0, 158.5, 151.5, 139.7, 133.9, 130.2, 122.0, 117.7, 115.3, 110.3, 108.5, 98.7, 69.5, 64.3, 56.2, 54.3 (2C), 36.7, 29.5 (2C); MS (*m*/*z*) [M + H]^+^ 405.66/407.65; HRMS *m*/*z* calcd. for C_21_H_26_N_2_O_4_Cl [M + H]^+^ 405.1581, found 405.1582; m.p. 140 °C; IR (neat, cm^−1^) ν_max_ 3439, 3302, 3213, 2921, 2852, 1695, 1599, 1449, 1240, 1229, 767, 697.

*4-Amino-5-chloro-N-[[1-[(3-hydroxyphenyl)methyl]-4-piperidyl]methyl]-2-methoxybenzamide* (**6**). Following procedure A with compound **14** (187 mg, 0.47 mmol). We obtained compound **6**, as a brown powder (97 mg, 51% isolated yield). ^1^H NMR (MeOD, 400 MHz) *δ* 7.79 (s, 1H, H_6_), 7.14 (td, 1H, *J* = 7.5, 1.2 Hz), 6.79 (m, 1H), 6.78 (m, 1H), 6.71 (m, 1H), 6.50 (s, 1H), 3.91 (s, 3H), 3.53 (s, 2H), 3.28 (d, 2H, *J* = 6.7 Hz), 3.02–2.96 (m, 2H), 2.16–2.08 (m, 2H), 1.78–1.71 (m, 2H), 1.65 (m, 1H), 1.42–1.28 (m, 2H); ^13^C NMR (MeOD, 100 MHz) *δ* 167.3, 159.4, 158.6, 150.2, 138.9, 133.1, 130.3, 122.1, 117.8, 115.6, 111.6, 111.5, 98.6, 64.0, 56.6, 54.3 (2C), 45.9, 37.1, 30.3 (2C); MS (*m*/*z*) [M + H]^+^ 404.64–406.64; HRMS (*m*/*z*) calcd. for C_21_H_27_N_3_NaO_3_ [M + H]^+^ 404.1741, found 404.1736; m.p. 92–105 °C; IR (neat, cm^−1^) ν_max_ 3391, 3204, 2928, 1627, 1592, 1544, 1462, 1253, 1211, 1148, 770.

#### 4.1.4. General Procedure for the Synthesis of Compounds (**7**–**9**)

Procedure B. To a stirred solution of *tert*-butyl piperidine-1-carboxylate derivative (1.0 equiv.) in DCM (20 mL/mmol) was added TFA (2 mL/mmol). The resulting mixture was stirred at room temperature for 1h. Removal of the solvent under vacuum afforded the crude product, which was directly engaged in the next step. The residue obtained (1.0 equiv) was dissolved in DMF (10 mL/mmol) and **12** (1.3 equiv) and K_2_CO_3_ (10.0 equiv) were added. The resulting mixture was stirred at 110 °C for 1 h, then concentrated in vacuo. Ethyl acetate was added, the organic layer was washed several times with brine, dried over MgSO_4_ and concentrated in vacuo. The crude product was purified by flash chromatography on silica gel column (DCM/MeOH, gradient 100:0 to 90:10) and concentrated under reduced pressure to afford alkylated compound.

*[3-[[4-[3-(4-Amino-5-chloro-2-methoxyphenyl)-3-oxopropyl]-1-piperidyl]methyl]phenyl] N-ethyl-N-methyl-carbamate* (**7**). Following procedure B with compound **12** (123 mg, 0.31 mmol). We obtained compound **7**, as a brown oil (80 mg, 50% isolated yield). ^1^H NMR (DMSO, 400 MHz, 363 K) *δ* 7.51 (s, 1H), 7.29 (t, 1H, *J* = 7.8 Hz), 7.12 (d, 1H, *J* = 7.8 Hz), 7.03 (s, 1H), 6.97 (dd, 1H, *J* = 7.8, 2.4 Hz), 6.51 (s, 1H), 5.84 (s, 2H), 3.81 (s, 3H), 3.45 (s, 2H), 3.39 (q, 2H, *J* = 7.1 Hz), 2.97 (s, 3H), 2.85–2.76 (m, 4H), 2.01–1.96 (m, 2H), 1.66–1.59 (m, 2H), 1.50 (q, 2H, *J* = 7.2 Hz), 1.28–1.12 (m, 6H); ^13^C NMR (DMSO, 100 MHz, 363 K) *δ* 196.9, 159.0, 153.6, 151.0, 149.1, 140.2, 130.5, 128.2, 124.7, 121.1, 119.4, 116.7, 109.2, 97.6, 61.5, 55.3, 52.8 (2C), 43.0, 41.1, 34.7, 33.2, 31.5 (2C), 30.6, 12.1; MS (*m*/*z*) [M + H]^+^ 488.75/490.74; HRMS (*m*/*z*) calcd. for C_26_H_34_ClN_3_O_4_ [M + H]^+^ 488.2316, found 488.2323; IR (neat, cm^−1^) ν_max_ 3439, 2918, 2852, 1713, 1621, 1589, 1250, 1167.

*[1-[[3-[Ethyl(methyl)carbamoyl]oxyphenyl]methyl]-4-piperidyl]methyl 4-amino-5-chloro-2-methoxybenzoate* (**8**). Following procedure B with compound **13** (143 mg, 0.36 mmol). We obtained compound **8**, as a colourless oil (86 mg, 46% isolated yield). ^1^H NMR (DMSO, 400 MHz, 363 K) *δ* 7.57 (s, 1H), 7.30 (t, 1H, *J* = 7.8 Hz), 7.13 (d, 1H, *J* = 7.8 Hz), 7.05 (t, 1H, *J* = 2.2 Hz), 6.98 (dd, 1H, *J* = 7.8, 2.2 Hz), 6.50 (s, 1H), 5.81 (br s, 2H), 4.03 (d, 2H, *J* = 5.8 Hz), 3.75 (s, 3H), 3.48 (s, 2H), 3.39 (q, 2H, *J* = 7.1 Hz), 2.97 (s, 3H), 2.87–2.79 (m, 2H), 2.06–1.96 (m, 2H), 1.73–7.63 (m, 3H), 1.42–1.26 (m, 2H), 1.17 (t, 1H, *J* = 7.1 Hz); ^13^C NMR (DMSO, 100 MHz, 363 K) *δ* 163.7, 159.3, 153.2, 151.1, 149.1, 140.0, 131.6, 128.2, 124.6, 121.1, 119. 4, 108.0, 107.8, 98.4, 67.4, 61.4, 55.5 (2c), 52.3, 43.0, 34.7, 33.2, 28.1 (2C), 12.1; MS (*m*/*z*) [M + H]^+^ 490.70/492.71; HRMS (*m*/*z*) calcd. for C_25_H_33_ClN_3_O_5_ [M + H]^+^ 490.2109, found 490.2106; IR (neat, cm^−1^) ν_max_ 3470, 3352, 3213, 2936, 2804, 2760, 1709, 1622, 1602, 1237, 1167, 694.

*[3-[[4-[[(4-Amino-5-chloro-2-methoxybenzoyl)amino]methyl]-1-piperidyl]methyl]phenyl] N-ethyl-N-methyl-carbamate* (**9**). Following procedure B with compound **14** (148 mg, 0.37 mmol). We obtained compound **9**, as a yellow oil (126 mg, 69% isolated yield). ^1^H NMR (DMSO, 400 MHz, 363 K) *δ* 7.72 (t, 1H, *J* = 6.1 Hz), 7.68 (s, 1H), 7.29 (t, 1H, *J* = 7.9 Hz), 7.12 (d, 1H, *J* = 7.9 Hz), 7.04 (s, 1H), 6.98 (dd, 1H, *J* = 7.9, 2.2 Hz), 6.53 5s, 1H), 5.61 (br s, 2H), 3.84 (s, 3H), 3.48 (s, 2H), 3.38 (q, 2H, *J* = 6.1 Hz), 3.20 (t, 2H, *J* = 6.1 Hz), 2.97 (s, 3H), 2.85–2.79 (m, 2H), 2.04–1.95 (m, 2H), 1.67–1.60 (m, 2H), 1.50 (m, 1H), 1.31–1.19 (m, 2H), 1.16 (t, 3H, *J* = 7.0 Hz); ^13^C NMR (DMSO, 100 MHz, 363 K) *δ* 16.3.4, 157.0, 153.2, 151.1, 147.7, 139.9, 131.0, 128.2, 124.7, 121.2, 119.5, 111.3, 109.0, 97.9, 61.4, 55.7, 52.4 (2C), 44.0, 43.1, 35.3, 33.3, 29.2 (2C), 12.1; MS (*m*/*z*) [M + H]^+^ 489.61/491.70; HRMS (*m*/*z*) calcd. for C_29_H_34_ClN_4_O_4_ [M + H]^+^ 489.2269, found 489.2268; IR (neat, cm^−1^) ν_max_ 3400, 2921, 1709, 1631, 1592, 1243, 1164, 688.

#### 4.1.5. General Procedure for the Preparation of Fumarate Salts (**19**–**20**)

Procedure C. To a stirred solution of basic compound (1.0 equiv) in iPrOH was added fumaric acid (0.95 equiv). The solution was refluxed for 1 h 15 min. The mixture was then concentrated in vacuo. The residue is triturated in Et_2_O and then filtrated to yield the fumarate salt.

*1-(4-Amino-5-chloro-2-methoxyphenyl)-3-[1-[(3-hydroxyphenyl)methyl]-4-piperidyl]propan-1-one fumaric acid salt* (**19**). Following procedure C with compound **4** (135 mg, 0.34 mmol). We obtained compound **19**, as a yellow powder (116 mg, 66% yield). ^1^H NMR (MeOD, 400 MHz) *δ* 7.65 (s, 1H), 7.26 (t, *J* = 8.1 Hz), 6.94–6.89 (m, 2H), 6.89–6.83 (m, 1H), 6.69 (s, 1H), 6.44 (s, 1H), 4.10 (s, 2H), 3.85 (s, 3H,), 3.43–3.35 (m, 2H), 2.99–2.90 (m, 2H), 2.90–2.78 (m, 2H), 2.00–1.87 (m, 2H), 1.66–1.51 (m, 3H), 1.51–1.38 (m, 2H); ^13^C NMR (MeOD, 100 MHz) *δ* 200.3, 172.6 (2C), 161.7, 159.3, 151.8, 136.6 (2C), 132.9, 132.7, 131.2, 122.9, 118.8, 117.7, 111.7, 98.0, 61.7, 56.1, 53.5, 41.3, 34.7, 31.6 (3C), 30.5 (2C); MS (*m*/*z*) [M + H]^+^ 403.60/405.61, HRMS *m*/*z* calcd. for C_22_H_28_N_2_O_3_Cl [M + H]^+^ 403.1788, found 403.1790; m.p. > 250 °C; IR (neat, cm^−1^) ν_max_ 333.1, 3207, 1622.2, 1583.6, 1454.4, 1361.8, 1215.2.

*[3-[[4-[3-(4-Amino-5-chloro-2-methoxyphenyl)-3-oxopropyl]-1-piperidyl]methyl]phenyl] N-ethyl-N-methyl-carbamate fumaric acid salt* (**20**). Following procedure C with compound **7** (135 mg, 0.28 mmol). We obtained compound **20**, as a white powder (114 mg, 68% yield). ^1^H NMR (MeOH, 400 MHz) δ 7.66 (s, 1H), 7.49 (t, *J* = 7.9 Hz, 1H), 7.36 (dt, *J* = 7.9, 1.3 Hz, 1H), 7.30 (t, *J* = 2.1 Hz, 1H), 7.23 (dd, *J* = 8.1, 2.1 Hz, 1H), 6.70 (s, 2H), 6.44 (s, 1H), 4.26 (s, 2H), 3.85 (s, 3H), 3.56–3.36 (m, 4H), 3.10 (s, 1.5 H, Rota A), 2.99 (s, 1.5 H, Rota B), 2.98–2.89 (m, 4H), 2.00–1.90 (m, 2H), 1.66–154 (m, 3H), 1.54–1.40 (m, 2H), 1.26 (t, *J* = 7.1 Hz, 1.5 H, Rota A), 1.18 (t, *J* = 7.1 Hz, 1.5 H, Rota B); ^13^C NMR (MeOH, 100 MHz) δ 200.1, 171.2, 161.7, 156.1 (Rota A), 156.0 (Rota B), 153.2, 151.8, 136.2 (2C), 132.9, 132.3 (Rota A), 132.2 (Rota B), 131.3, 129.3, 125.9 (Rota A), 125.8 (Rota B), 124.6 (Rota A), 124.5 (Rota B), 117.7, 111.7, 98.0, 60.8, 56.1, 53.5 (2C), 45.3 (Rota A), 45.2 (Rota B), 41.2, 34.6 (Rota A), 34.3 (Rota B), 34.5, 31.5, 30.3 (2C), 13.4 (Rota A), 12.6 (Rota B); MS *m*/*z* [M + H]^+^ 488.75/490.74; HRMS *m*/*z* calcd. for C_26_H_35_N_3_O_4_Cl [M + H]^+^ 488.2316, found 488.2318; m.p. > 250 °C; IR (neat, cm^−1^) ν_max_ 3439, 2918, 2852, 1713, 1621, 1589, 1250, 1167.

### 4.2. In Silico Study

The initial model of compound **7** was built from donecopride X-ray structure [5], and its protonation state at pH 7.4 was predicted using standard tools of the ChemAxon Package (http://www.chemaxon.com/). The majority microspecies protonated on piperidine nitrogen at this pH was used for docking studies.

The crystallographic coordinates of human acetylcholinesterase used in this study were obtained from X-ray structure of the donepezil/AChE complex (PDB ID 4EY7, a structure refined to 2.35 Å with an R factor of 17.7%). Structures of human acetylcholinesterase in complex with pharmacologically important ligands [13]. The AChE amino acid protonation state was checked before the docking study using the ProPKA software and the proposed protonation for Glu202 was applied.

The docking of the compound into the AChE was carried out with the GOLD program (v5.7.2, Cambridge Crystallographic Data Center, CCDC) using the default parameters [23,24]. This program applies a genetic algorithm to explore conformational spaces and ligand binding modes. To evaluate the proposed ligand positions, the ChemPLP fitness function was used. The binding site in the AChE model was defined as a 7 Å sphere from the co-crystallized donepezil ligand, and a water molecule interacting with a protonated piperidine ring of donepezil was conserved during the docking (residue number 931). The Ser203 side chain was kept flexible and a hydrogen bond constraint between Ser203 side chain and carbonyl oxygen was applied during the docking.

### 4.3. Biological Evaluation

#### 4.3.1. In Vitro Tests of AChE Inhibitory Activity

The inhibitory capacity of compounds on AChE biological activity was evaluated through the use of the spectrometric method of Ellman [14]. Acetylthiocholine iodide and 5,5-dithiobis-(2-nitrobenzoic) acid (DTNB) were purchased from Sigma Aldrich. AChE from human erythrocytes (buffered aqueous solution, ≥500 units/mg protein (BCA), Sigma Aldrich, St. Louis, MO, USA) was diluted in 20 mM HEPES buffer pH 8, 0.1% Triton X-100 such as to have enzyme solution with 0.25 unit/mL enzyme activity. In the procedure, 100 μL of 0.3 mM DTNB dissolved in a phosphate buffer with pH 7.4 were added into the 96-well plates followed by 50 μL of the test compound solution and 50 μL of enzyme (0.05 U final). After 5 min of preincubation at 25 °C, the reaction was then initiated by the injection of 50 μL of 10 mM acetylthiocholine iodide solution. The hydrolysis of acetylthiocholine was monitored by the formation of yellow 5-thio-2-nitrobenzoate anion as the result of the reaction of DTNB with thiocholine, released by the enzymatic hydrolysis of acetylthiocholine, at a wavelength of 412 nm using a 96-well microplate plate reader (BioTek, Synergy 2, Winooski, VT, USA). Test compounds were dissolved in analytical grade DMSO. Donepezil (DPZ) and Rivastigmine were used as a reference standard. The rate of absorbance increase at 412 nm was followed every minute for 10 min. Assays were performed with a blank containing all components except acetylthiocholine, in order to account for non-enzymatic reactions. The reaction slopes were compared and the percent inhibition due to the presence of test compounds was calculated by the following expression: 100 − (*v_i_*/*v_0_* × 100), where *v_i_* is the rate calculated in the presence of inhibitor and *v*_0_ is the enzyme activity. The first screening of AChE activity was carried out at a 10^−6^ concentration of compounds under study. For the compounds with significant inhibition (≥50%), IC_50_ values were determined graphically by plotting the % inhibition versus the logarithm of six inhibitor concentrations in the assay solution using the GraphPad Prism 6 software.

#### 4.3.2. Kinetic Study for AChE Inhibition

To try to clarify the mechanism of action of **19** and **20**, reciprocal plots of 1/velocity vs. 1/[substrate] were constructed at different concentrations of the substrate acetylthiocholine iodide (0.01–1 mM) by using the spectrometric method by Ellman et al. [14]. Four concentrations of **19** were selected for the studies: 10^−6^, 10^−7^, 10^−8^ and 10^−9^ M for the kinetic analysis of AChE inhibition. Four concentrations of **20** were selected for the studies: 10^−5^, 10^−6^, 10^−7^ and 10^−8^ M for the kinetic analysis of AChE inhibition. The plots were assessed by a weighted least squares analysis that assumed the variance of velocity (ν) to be a constant percentage of ν for the entire dataset. Slopes of these reciprocal plots were then plotted against the concentration of **19** and **20** in a weighted analysis.

#### 4.3.3. Pharmacological Characterization of Drugs on Human 5-HT_4_R

For competition studies, 2.5 µg of proteins (5-HT_4(b)_ membrane preparations, HTS110M, Eurofins. Eurofins’ 5-HT_4(b)_ membrane preparations are crude membrane preparations made from their proprietary stable recombinant cell lines to ensure high-level of GPCR surface expression.) were incubated in duplicate at 25 °C for 60 min in the absence or the presence of 10^−6^ or 10^−8^M of each drug (**9** was used as a reference standard) and 0.2 nM [^3^H]-GR113808 (NET 1152, Perkin Elmer) in 25 mM Tris buffer (pH 7.4, 25 °C). At the end of the incubation, homogenates were filtered through Whatman GF/C filters (Alpha Biotech) pre-soaked with 0.5% polyethylenimine using a Brandel cell harvester. Filters were subsequently washed three times with 1 mL of ice-cold 25 mM Tris buffer (pH 7.4, 4 °C). Non-specific binding was evaluated in parallel in the presence of 30 µM serotonin. For some of these compounds, affinity constants were calculated from five-point inhibition curves using the GraphPad Prism 6 software and expressed as Ki ± SD.

#### 4.3.4. Determination of cAMP Production

COS-7 cells were grown in Dulbecco’s modified Eagle medium (DMEM) supplemented with 10% dialyzed fetal calf serum (dFCS) and antibiotics. Cells were transiently transfected by electroporation with plasmids encoding HA-tagged 5-HT_4_R (100 ng/10^6^ cells), then seeded in 96-well plates (16,000 cells/well). Twenty-four hr after transfection, cells were exposed to the indicated concentrations of 5-HT_4_R ligands in the presence of 0.1 mM of the phosphodiesterase inhibitor RO-20-1724, at 37 °C in 100 µL of HBS (20 mM HEPES; 150 mM NaCl; 4.2 mM KCl; 0.9 mM CaCl_2_; 0.5 mM MgCl_2_; 0.1% glucose; 0.1% BSA). After 10 min, cells were then lysed by the addition of the same volume of Triton-X100 (0.1%). Quantification of cAMP production was performed by HTRF^®^ using the cAMP Dynamic kit (Cisbio Bioassays, Codolet, France) according to the manufacturer’s instructions.

#### 4.3.5. Parallel Artificial Membrane Permeability Assay

The parallel artificial membrane permeability assay (PAMPA) blood–brain barrier (BBB) experiments were conducted using the Pampa Explorer Kit (Pion Inc., Billerica, MA, USA) according to the protocol provided by the manufacturer. Briefly, the donecopride fumarate solution (20 mM in DMSO) was diluted in Prisma HT buffer (pH 7.4; pION) to 100 μM; 200 μL this solution (*n* = 6) was added to the donor plate (P/N 110,243). Five microliters BBB-1 Lipid (P/N 110,672) was used to coat the membrane filter of the acceptor plate (P/N 110,243). Two hundred microliters Brain Sink Buffer (P/N 110,674) was added to each well of the acceptor plate. The PAMPA sandwich was assembled and allowed to incubate at room temperature for 4 h without stirring. The sandwich was then separated, and the UV-visible spectra were measured for both the donor and receiver wells with the microplate reader (Tecan Infinit M200). The −logPe and Pe (PAMPA effective permeability coefficient) were calculated by the PAMPA Explorer software version 3.7 (pION) for studied compounds. Corticosterone (−logPe = 4.76, Pe = 17.2 × 10^−6^ cm/s) and theophylline (−log Pe = 6.44, Pe = 0.40 × 10^−6^ cm/s) were used as positive and negative references, respectively.

#### 4.3.6. AChE-Dependent Decarbamoylation

The HPLC analyses were performed using an Agilent pump 1290, an autosampler 1290 and a diode array UV detector 1260 (Agilent technologies, Santa Clara, CA, USA). A reversed phase column C18 (Zorbax1 Eclipse Plus RRHD, 1.8 µm, 2.1 × 50 mm, Agilent) has been used for all components. A binary pump was used and the mobile phase was always composed of a mixture of (A) water (0.1% formic acid) and (B) acetonitrile (0.1% formic acid). Solutions of compound **19** and **20** were prepared in a PBS buffer (pH 7.4). AChE from *Electrophorus electricus* (buffered aqueous solution, ≥500 units/mg protein (BCA), Sigma Aldrich) was diluted in PBS buffer pH 7.4, such as to have enzyme solution with 250 unit/mL enzyme activity. Solution was prepared by addition of 250 μL of the abovementioned enzyme stock solution to 5 μL of solution of compounds 19 or 20 at 255 µM. The mixture was stirred during 24 h at rt. The mixture was extracted by 300 µL of EtOAc, and 250 µL of the organic layer was reduced by nitrogen flow. The crude product was submerged in 50 µL of ACN, then analyzed by UPLC and LC-MS.

#### 4.3.7. In Vivo Biological Studies

*Animals.* Adult male NMRI mice (3 months old, weighing 35–40 g) from Janvier labs (Le Genest-Saint-Isle, France) were used to perform experiments. Mice were housed by ten in standard polycarbonate cages in standard controlled conditions (22 ± 2 °C, 55 ± 10% humidity) with a reversed 12 h light/dark cycle (light on at 7 pm). Food and water were available ad libitum in the home cage. All experiments were conducted (between 9 am and 3 pm) during the active–dark phase of the cycle and were in agreement with the European Directives and French law on animal experimentation (personal authorization n° 14–17 for MB and 14–60 for TF).

*CNS-activity and acute toxicity test.* Behavioral and neurological changes induced by graded doses (1, 10, 100 mg/kg) of the tested derivatives were evaluated in mice, in groups of four, by a standardized observation technique at different times (30 min, 3 and 24 h) after intraperitoneal administration [25]. Major changes of behavioral data (for example, hypo- or hyperactivity, ataxia, tremors, convulsion, etc.) were noted in comparison to the control group. The approximate DL_50_ of the compounds were also calculated through the quantification of mortality after 24 h. Amphetamine (2 mg/kg) and chlorpromazine (10 mg/kg) were used as the stimulant and depressive references, respectively.

*Locomotor activity.* The locomotion of mice was measured using an actimeter (Imetronic^®^, Pessac, France) through infrared detection. Eight individual removable polycarbonate cages (21 cm length, 7 cm wide and 12 cm high), in which each mouse was placed, were used in the actimeter. Locomotor activity was measured by recording the number of interruptions of beams of the red light over a period of 30 min through a recording system attached to the actimeter. Compound **20** was tested at 1, 3 and 10 mg/kg. Chlorpromazine (3 mg/kg) were used as depressive reference [26].

*Spatial working memory.* The antiamnesic activity of the tested compounds was evaluated by a reversal of deficit on spontaneous alternation behavior in the Y maze test [27]. Deficits were pharmacologically induced either by scopolamine (SCOP, 0.5 mg/kg) or dizocilpine (MK801, 0.1 mg/kg). The Y maze made of grey plastic consisted of three equally-spaced arms (21-cm long, 7-cm wide with walls 15-cm high). The mouse was placed at the end of one of the arms and allowed to move freely through the maze during a 5 min session, while the sequence of arm entries was recorded by an observer. An arm entry was scored when all four feet crossed into the arm. An alternation was defined as entries into all three arms on a consecutive occasion. The number of possible alternations is thus the total number of arm entries minus two; the percentage of alternation was calculated as (actual alternation/possible alternation) 100. Compound **20** was tested at 1, 3 and 10 mg/kg.

*Pharmacological treatments.* Amphetamine (+)-α-Methylphenethylamine hemisulfate, chlorpromazine hydrochloride, MK801 hydrogen maleate and scopolamine hydrobromide were purchased from Sigma (France). All pharmacological compounds were dissolved in NaCl 0.9%, used as vehicle. Besides, all were administered IP 30 min before tests, except for scopolamine and MK801, which were subcutaneously administered 20 min before spontaneous alternation test.

*Statistical analysis.* Results were expressed as mean ± SD and were analyzed by one-way analysis of variance (ANOVA), with Statview^®^ software (Abacus Concepts, Berkeley, CA, USA). In case of significance, a SNK (Student-Newman-Keuls) post hoc test was realized. Additionally, for the spontaneous alternation test, the percentage of alternation was compared to a theoretical 50% value (random alternation) by a univariate *t*-test. Differences were considered as statistically significant if the *p* value was strictly under 0.05.

## 5. Conclusions

We succeeded in designing and synthesizing a pleiotropic prodrug which benefits from a carbamate structure **20** to easily cross the BBB and covalently bind to central AChE. Upon hydrolysis by the enzyme, **20** releases a phenol compound **4** which, contrarily to **20**, acts both as a DBS AChEI and as a 5-HT_4_R partial agonist. With this unique in vitro profile, **20** displayed, in vivo at a low dose of 1 mg/kg, antiamnesic effects towards cognitive deficits, resulting from either cholinergic or glutamatergic neurotransmission impairments in mice. These results seem to support conjecture that this pleiotropic prodrug is both peripherally safe and shows great efficiency in AD pathogenesis and symptoms.

## Figures and Tables

**Table 1:**
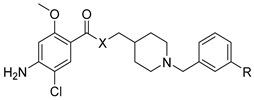
(*h*)AChE inhibitory Activity (% inhibition or IC_50_) and (*h*)5-HT_4_R affinity (% inhibition or K*i*).

Compound	X	R	(*h*)AChE IC_50_ (nM) or % Inhibition at 10^−6^ M	(*h*)5-HT_4_R K*i* (nM) or % Inhibition at 10^−8^ M
Donepezil	-	-	7.0 ± 1.5	-
Rivastigmine	-	-	4.150 ± 160 ^1^	-
RS67333	-	-	-	5.1 ± 0.5 ^2^
**4**	CH_2_	OH	148.6 ± 34.9	5.1 ± 1.1
**7**	CH_2_	OCON(Me)Et	4151 ± 794	3%
**5**	O	OH	11%	0.6 ± 0.4
**8**	O	OCON(Me)Et	16,290 ± 240	23%
**6**	NH	OH	4%	5.8 ± 1.2
**9**	NH	OCON(Me)Et	10,077 ± 2,988	13%
**19**	CH_2_	OH, fumaric salt	72.0 ± 1.4	6.9 ± 1.2
**20**	CH_2_	OCON(Me)Et, fumaric salt	6070 ± 404	16%

IC_50_ values are expressed as mean ± standard error of the mean (SEM) of at least two experiments; K*i* values are expressed as mean ± standard error of the mean (SEM) of at least three experiments; ^1^ Data from ref. [16]; ^2^ Data from ref. [17].

**Table 2:** (*h*)5-HT_4(a)_ pharmacological profile of RS67333 and compound **19**. 5-HT was used as a control 5-HT_4_R agonist.

Compound	Log(EC_50_) ^1^	% Control Agonist Response ^2^	Profile
RS67333	−8.8 ± 0.2	48.7 ± 5.2	Partial agonist
**19**	−9.90 ± 0.0	59.0 ± 3.9	Partial agonist

^1^ Log(EC_50_) values are expressed as mean ± standard error of the mean (SEM) of at least two (RS67333) or three experiments (**19**); ^2^ % Control agonist response values are expressed as mean ± standard error of the mean (SEM) of at least two experiments.

**Table 3:** logPe represents the PAMPA effective permeability coefficient results for **20**.

Compound	LogPe
Corticosterone	−4.84 ± 0.02
Theophylline	−6.52 ± 0.05
**20**	−4.39 ± 0.12

**Table 4:** Pharmacological and toxicological properties of **20**.

Compound	Dose (mg/kg)	LD_50_ (mg/kg)	Symptoms (Subtoxic Doses)
**20**	1–10–100	>100	1–10, no symptoms
			100, tremors, hypoactivity full recovery after 24h
Amphetamine ^1^	2	-	Hyperactivity, exophtalmy, irritability
Chlorpromazine ^1^	10	-	Hypoactivity, ataxia, sleep

^1^ Amphetamine (2 mg/kg), chlorpromazine (10 mg/kg) were used as stimulant and depressive references, respectively.
